# Do habits always override intentions? Pitting unhealthy snacking habits against snack-avoidance intentions

**DOI:** 10.1186/s40359-015-0065-4

**Published:** 2015-03-24

**Authors:** Benjamin Gardner, Sharon Corbridge, Laura McGowan

**Affiliations:** Health Behaviour Research Centre, Department of Epidemiology and Public Health, University College London, London, UK; Current affiliation: Department of Psychology, Institute of Psychiatry, Psychology and Neuroscience, King’s College London, 9th Floor, Capital House, 42 Weston Street, London, SE1 3QD UK; Current affiliation: Institute for Global Food Security, Northern Ireland Technology Centre, Queen’s University Belfast, 18-30 Malone Road, Room 02.024, Belfast, BT9 5BN UK

**Keywords:** Habit, Automaticity, Reasoned action, Health behaviour, Diet, Snacking

## Abstract

**Background:**

Habit is defined as a process whereby an impulse towards behaviour is automatically initiated upon encountering a setting in which the behaviour has been performed in the past. A central tenet of habit theory is that habit overrides intentional tendencies in directing behaviour, such that as habit strength increases, intention becomes less predictive of behaviour. Yet, evidence of this effect has been methodologically limited by modelling the impact of positively-correlated habits and intentions. This study sought to test the effect of habits for unhealthy snacking on the relationship between intentions *to avoid* unhealthy snacks and snack intake.

**Methods:**

Methods were chosen to match those used in studies that have shown habit-intention interactions. 239 adults completed valid and reliable measures of habitual snacking and intention to avoid snacking at baseline, and a self-report measure of snack intake two weeks later. Data were analysed using multiple regression.

**Results:**

While both habit and intention independently predicted snack intake, no interaction between habit and intention was found.

**Conclusions:**

No support was found for the expected moderating impact of habit on the intention-behaviour relationship, indicating that individuals with intentions can act on those intentions despite having habits. Previous evidence of a habit-intention interaction effect may be unreliable. A growing literature indicates that habitual tendencies can be inhibited, albeit with difficulty. Habits and intentions may vary in the influence they exert over discrete behaviour instances. While the aggregation of behaviours across instances and individuals used in our study reflects the dominant methodology in habit research, it precludes examination of effects of in-situ habits and intentions. More sophisticated data collection and analysis methods may be needed to better understand potential habit-intention interactions.

**Electronic supplementary material:**

The online version of this article (doi:10.1186/s40359-015-0065-4) contains supplementary material, which is available to authorized users.

## Background

Behaviour change interventions have often had limited success because short-term changes erode over the longer-term (e.g. Jeffery *et al*. 1990). When a health behaviour change intervention is withdrawn, enthusiasm for the healthy behaviour is often lost, and participants lapse back into unhealthy behavioural patterns. One mechanism that is attracting attention as a means of maintaining behaviour change over the long-term is ‘habit’. Habit has been defined as a process whereby a situation automatically generates an impulse towards doing an action that has been repeatedly performed in that situation; ‘habitual behaviours’ are actions controlled by this process (Gardner 2015a). Habitual behaviours are learned through a process of ‘context-dependent repetition’ (Lally et al. 2010). Each performance of a given behaviour in a given setting reinforces a mental association between the behaviour and the setting, such that alternative actions become less accessible in memory, and the chosen behaviour becomes the ‘default’ option (Wood and Neal 2009). Habit is said to have formed when encountering the situation becomes sufficient to activate an impulse towards the associated behaviour which can subsequently control behaviour without intention, awareness, or conscious control.

Habit is often defined in contrast with reasoned, deliberative concepts such as conscious intentions. Dual process models portray two pathways to action which may be engaged upon encountering situational cues (Borland 2013; Strack and Deutsch 2004). The reflective pathway involves thoughtful deliberation over the utility of available behavioural options, culminating in the formation of an intention to act. Habit sits on a parallel, impulsive pathway, such that perception of cues activates low-level associative responses, without conscious awareness. Whereas the generation of behaviour via the reflective system is cognitively effortful, the impulsive pathway directs behaviour quickly and with minimal effort. It is theorised that, where habits and intentions conflict, the impulsive system will generate habitual behaviour more rapidly than the reflective system can instigate counterhabitual intentions (e.g. Triandis 1977). Thus, habit is thought to moderate the intention-behaviour relationship, such that intentions are less predictive of behaviour where habit is strong (Triandis 1977).

The hypothesised moderating effect of habit on the intention-behaviour relation underpins current interest in habit as a means of maintaining behaviour change. Commentators have reasoned that, if a healthy behaviour can be made habitual, it will be less prone to disruption when motivation wanes (Verplanken and Wood 2006). Behaviour change interventions should thus seek to promote healthy habits, as a means of shielding the new behaviour against losses in motivation, which might otherwise result in a long-term reversal of short-term behaviour gains (Rothman et al. 2009). At first glance, evidence for this effect appears compelling: for example, a meta-analysis of applications of habit to dietary and physical activity domains found that eight of nine tests of moderation showed results in line with this hypothesis (Gardner et al. 2011). Yet, some more recent studies have found moderation in the opposite direction, such that habits *strengthen* the relationship between intention and behaviour (e.g. de Bruijn et al. 2012; Gardner et al. 2012a), and some tests have found no moderation (e.g. Murtagh et al. 2012).

There are methodological reasons to question the validity of evidence that habit overrides the impact of intentions on behaviour. Studies tend to infer moderation by modelling the impact of intention on behaviour at different levels of habit. Yet, as a recent review showed (Gardner 2015a), most studies have measured habit and intention concurrently (e.g. habit for driving to work, intention to drive to work; Gardner 2009). Habits arise through repeated performance of an intended action (Lally *et al*. 2010; Tobias 2009), and so, unless individuals have been exposed to a natural or purposive intervention, habits should be expected to correlate with intentions. Indeed, studies of concurrent habits and intentions tend to show high habit-intention correlations (e.g. Gardner 2009; van Bree et al. 2013). Forecasts of behaviour where habit is strong and intentions are weak, and vice versa, thus lack ecological validity, as there are unlikely to be many participants with such cognition patterns within the sample. Gardner (2015a) argues that a weak intention to do a given behaviour (e.g. to eat unhealthy snacks) cannot reliably be interpreted as a strong intention to perform an alternative (to eat healthy snacks) or to inhibit the behaviour (e.g. to avoid eating unhealthy snacks). For these reasons, the potential interaction between habit and intention should be investigated by measuring conflicting intentions and habits (e.g. intention to avoid eating unhealthily versus unhealthy eating habits). Two studies have adopted this approach, in the domains of unhealthy eating (Gardner et al. 2012b) and active travel (Murtagh et al. 2012), and neither found moderation.

This study was designed to provide further evidence around the theorised moderating effect of habit on the intention-behaviour relationship, where habits and intentions conflict. We focused on unhealthy eating, as a setting in which habits (for unhealthy snacking) could reasonably be expected to be incongruent with intentions (to avoid eating unhealthy snacks). To ensure comparability with previous studies of the habit-intention interaction, we adopted the dominant methods used in those studies, using a questionnaire survey design, with validated measures of habit and intention taken at baseline, and self-reported behaviour at a later point (e.g. Gardner 2015a; Gardner et al. 2011).

Two hypotheses were tested. The habit process generates behaviour impulsively and automatically, and so more strongly habitual behaviours should be elicited more often than less habitual behaviours. Hence, habit tends to correlate moderately-to-strongly with behaviour (Gardner et al. 2011). To ensure the comparability of our data to previous datasets, our first, preliminary hypothesis was that:

- *Hypothesis 1.* Unhealthy snacking habits will correlate with unhealthy snack intake.

Our main hypothesis, based on the theorised impact of counterintentional habits on the intention-behaviour relationship (Triandis 1977), was:

- *Hypothesis 2.* Unhealthy snacking habits will override intentions to avoid eating unhealthy snacks, such that, where unhealthy snacking habits are stronger, snack avoidance intentions will have less impact on behaviour.

## Methods

### Design and procedure

A prospective design was used. Participants completed an online survey, in which they provided measures of habit and intention and their email address at Time 1 (T1; Additional file 1), and two weeks later (time 2; T2) were sent an email requesting measures of behaviour over the preceding two weeks (Additional file 2). Questionnaires were successfully piloted on a sample of 10 participants for comprehension.

Participants were recruited via internal emails containing a link to the T1 questionnaire, which was sent with employers’ consent to employees of a UK financial services organisation. An invitation to participate was also posted in a staff newsletter within a UK university, and recruitment adverts were posted on social media websites. Participants received entry into a £50 voucher prize draw on completion of T1 and T2 questionnaires. On the survey website, prior to questionnaire completion, participants were informed that beginning to complete the questionnaire would be taken to indicate consent to participate. Approval was gained from the UCL Research Ethics Committee (ref 4538/001).

### Participants

Of 277 participants responding to the T1 questionnaire, 250 (90%) completed the T2 measure. Data were excluded from nine participants who gave incomplete responses, one participant who did not indicate their age, and one participant who gave measures of their total dietary intake rather than snack intake.

Our final sample comprised 239 participants who completed measures at both T1 and T2, representing 86% of T1 responders. No differences were found between the final sample and those who only completed baseline measures in terms of demographics or baseline predictor variables. Participant characteristics are detailed in Table 1. Participants were most typically female, White British, employed, educated to degree level or higher, and/or home-owners. Mean age was 41.8 years (SD 11.30), and mean body mass index (BMI; i.e., weight in kilograms divided by height in metres squared) was 25.3 kg/m^2^ (SD 5.59).

**Table 1:** **Participant characteristics**

**Gender**		***n***	***%***
	Male	53	22.2%
	Female	186	77.8%
**Age**		***Mean***	***SD***
*Range: 18-67*		41.77	11.30
**Ethnic Group**		***n***	***%***
	White-British	182	76.8%
	White-Irish	5	2.1%
	White-Other	39	16.5%
	Black-Caribbean	1	0.4%
	Black-Other	1	0.4%
	Bangladeshi	2	0.8%
	Asian Other	3	1.3.%
	Chinese	4	1.7%
**Employment**		***n***	***%***
	Employed	214	91.1%
	Unemployed	21	8.9%
**Education**		***n***	***%***
	No educational qualifications	2	0.9%
	CSE, GCSE or ‘O’ Levels	26	11.1%
	Vocational qualifications	16	6.8%
	‘A’ or ‘AS’ Level/ Higher School Certificate	32	13.6%
	Undergraduate Degree	80	34.0%
	Postgraduate qualification (e.g. Masters, PhD)	79	33.6%
**Home Ownership**		***n***	***%***
	Home owner	155	64.9%
	Private tenant	66	27.6%
	Council tenant	8	3.3%
	Living with parent/relative	10	4.2%
**BMI (kg/m** ^**2**^ **)**		***Mean***	***SD***
*Range: 17.32-48.48*		25.28	5.59
		***n***	***%***
	Healthy Weight	136	57.9%
	Overweight	69	29.4%
	Obese	30	12.8%

SD, standard deviation; BMI, body mass index; Healthy weight = BMI ≥18.5 < 25 kg/m^2^, Overweight = BMI ≥25 < 30 kg/m^2^, Obese = BMI ≥30 kg/m^2^.

A conservative *a priori* power calculation, conducted using G*Power (version 3.1.5; Faul et al. 2007) and based on detecting a small effect size (*f*^*2*^ = 0.1) for a regression analysis of up to 12 predictors, indicated a required sample of 230 to achieve power of 0.90 where p ≤ 0.05.

### Materials

The habit-intention interaction is most commonly tested using multiple regression models in which the predictive power of a habit-intention interaction variable is tested (Gardner 2015a; Gardner *et al*. 2011). Studies showing habit to moderate the intention-behaviour relationship have variously additionally controlled for variables drawn from the Theory of Planned Behaviour (TPB; Ajzen 1991; i.e. attitudes, subjective norms and perceived behavioural control [PBC]) and demographics. Hence, our baseline questionnaire featured measures of habit, intention, TPB variables, and demographics. The mean of multi-item measures was used to arrive at a single score for each construct. Items for all scales were reverse coded where appropriate so that higher scores reflected greater value of that construct.

#### Demographics

Gender, age, height and weight (to allow calculation of BMI), ethnic group, employment status, highest level of education and home ownership status were self-reported. BMI was categorised into healthy weight (BMI ≥18.5 < 25 kg/m^2^), overweight (BMI ≥25 < 30 kg/m^2^) and obese (BMI ≥ 30 kg/m^2^) for sample characterisation purposes only.

#### Habit

Habit was measured using an automaticity specific subscale of the Self-Report Habit Index (Verplanken and Orbell 2003), the Self-Report Behavioural Automaticity Index (Gardner et al. 2012b), which has been shown to have predictive and construct validity while retaining strong convergent validity with its parent scale. Four items followed a stem (‘eating unhealthy snacks is something…’): ‘…I do automatically', ‘…I do without having to consciously remember', ‘…I do without thinking’, ‘…I start doing before I realize I’m doing it’ (1 [*strongly disagree*] – 7 [*strongly agree*]; α = .93).

#### Intention and TPB variables

TPB variables towards *avoiding* eating unhealthy snacks were measured using scales recommended by Ajzen (2006), as adapted to unhealthy snacking behaviour (Churchill et al. 2008). Responses were given on a seven-point scale from 1 (*disagree strongly*) to 7 (*agree strongly*) unless otherwise stated.

*Intention* was measured using three items (‘I intend to avoid eating unhealthy snacks over the next two weeks’, ‘I want to avoid eating unhealthy snacks over the next two weeks', ‘I expect to avoid eating unhealthy snacks over the next two weeks’; α = .90). *Attitudes* were measured by two items (e.g. ‘My attitude towards avoiding eating unhealthy snacks over the next two weeks is…’: 1 [*extremely negative*] – 7 [*extremely positive*]; α = .83). Two items measured *subjective norms* (e.g. ‘People who are important to me think I should avoid eating unhealthy snacks over the next two weeks’; α = .71). Two items measured *PBC* (‘I have complete control over whether I avoid eating unhealthy snacks over the next two weeks’; α = .85).

#### Unhealthy snack intake (behaviour)

At T2 snack intake was measured using a pre-defined food frequency questionnaire for 21 snack foods. This was compiled from a pilot study in which a group of 20 adults listed the 10 foods they snacked on most frequently. The questionnaire deliberately did not refer to ‘healthy’ or ‘unhealthy’ snacks, but rather snacks in general, to avoid responses being influenced by differences in perceptions or knowledge of what constitutes a healthy or unhealthy snack. The resulting list of 21 snack foods represented the top 40% of snacks cited by the pilot sample.

Participants reported the frequency of consuming each snack food over the past two weeks, from *‘not at all’* (1) to *‘three or more times a day’* (7) and the typical portion size consumed from ‘*none*’ (1) to ‘*extra large’* (5) when compared to a provided example of a medium sized serving. For analysis purposes, snack foods were categorised as unhealthy or not based on nutrient profiling, where foods are classified by researchers depending on their nutritional composition and relationship to disease prevention and health promotion (Department of Health, UK 2011) (see Table 2). Fourteen of the 21 snack foods were classified as unhealthy and from this, an unhealthy snack intake variable was generated. Following Campbell *et al.* (2007) and McGowan et al. (2012), responses were assigned a score reflecting the average number of servings per day (0, 0.14, 0.29, 0.5, 1, 2 or 3), which was weighted according to portion size (0.5 for small, 1 for medium, 2 for large, 3 for extra-large). The resulting score was multiplied by 14 (i.e., number of days within the two-week period) to provide a total unhealthy snack intake score. The resultant scale was log-transformed to reduce observed skewness.

**Table 2:** **Nutrient Profiling Assessment of the 21 snack foods featured in the food frequency questionnaire**

***Snack Food***	***Assessed as unhealthy?***
Fresh Fruit	No
Dried Fruit	Yes
Chocolate	Yes
* Crisps *(USA: potato chips)*	Yes
Nuts and seeds (unsalted)	No
Nuts (salted, flavoured or coated)	Yes
* Biscuits *(USA: cookies)* – plain	Yes
* Biscuits *(USA: cookies)* – chocolate or cream	Yes
* Crackers and savoury biscuits *(USA: cookies)*	Yes
Breadsticks, oatcakes, rice cakes, pretzels	Yes
Rice cakes	No
Cheese (cheddar)	Yes
Toast or bread	No
Butter or margarine	Yes
Cakes and sweet pastries	Yes
Yogurt	No
Raw vegetables	No
Dips (eg; houmous or salsa)	No
Sweets *(USA: candy)*	Yes
Savoury pastries	Yes
Cereal bars	Yes

Whether foods were unhealthy or not was judged according to nutrient profiling assessment. * American (US) equivalents are provided for clarity.

### Analysis

Analyses were performed using SPSS (IBM Corp. Version 21.0. Armonk, NY). Associations between variables were assessed via Pearson correlations. A multiple regression analysis was used to predict perceived unhealthy snack intake, which is standard analytic procedure for testing habit-intention interactions (Gardner et al. 2011). Known demographic covariates of unhealthy dietary intake (age, gender, BMI, education [dichotomised into ‘degree level or higher’ vs all other education]; Public Health England and Food Standards Agency 2014; Kong *et al.*^2011^; Jeffery et al. 1991) were controlled at each step. Intention was entered as a predictor at the first step, habit at the second step, and an interaction term composed of means-centred habit x intention scores at the third step.

To determine whether results were influenced by covariates, two sensitivity analyses were run. The first excluded demographics, because the TPB predicts should have an influence on behaviour that is mediated by cognitions (Ajzen 1991). Some previous studies showing habit to override intentions in determining behaviour have controlled for hypothesised predictors of intention, as drawn from the TPB (e.g. de Bruijn 2010; de Bruijn et al. 2008). Hence, the second analysis included TPB variables (attitude, subjective norms, PBC), but excluded demographics.

## Results

### Correlations

#### Demographic covariates

Age was correlated with snacking intentions (see Table 3): older participants (r = .17, p = .01) had stronger intentions to avoid unhealthy snacks. Those with higher educational status reported weaker unhealthy snacking habits (r = −.18, p = .005).

**Table 3:** **Bivariate associations between demographics, intention, habit and unhealthy snack intake** ***(n = 228)***

	***1.***	***2.***	***3.***	***4.***	***5.***	***6.***
1. Gender						
2. Age	-.20**					
3. Education	.12	-.15*				
4. BMI	-.11	.20**	-.23**			
5. Intention *to avoid* eating unhealthy snacks	.11	.17**	-.12	.12		
6. Habit *for eating* unhealthy snacks	-.06	-.11	-.18**	.24***	-.18**	
7. Unhealthy snack intake	-.13	.03	-.04	.11	-.21**	.27***

*p < .05, **p < .01, ***p < .001.

Gender scored as 1 = male, 2 = female. Education scored as 1 = compulsory education OR vocational, A or AS level, 2 = undergraduate degree or higher.

#### Intention, habit and unhealthy snack intake

Intention to avoid unhealthy snacking was inversely correlated with unhealthy snacking habits (r = −.18, p = .005) and unhealthy snack intake (r = −.21, p = .002). Unhealthy snacking habit was positively correlated with unhealthy snacking intake (r = .27, p < .001), supporting Hypothesis 1.

### Intention and habit as predictors of unhealthy snack intake

As Table 4 shows, intention predicted snack intake at Step 1 (β = −.22, p = .001; R^2^ = .07, Model F [5,223] = 3.38, p = .006). At Step 2, intention (β = −.17, p = .01) and habit (β = .23, p = .001; R^2^ = .11, F change = 11.05, p = .001) were predictive. Adding the intention x habit interaction term at step 3 did not improve the model (R^2^ = .12, F change = 1.60, p = .21), and the interaction term had no impact on snack intake (β = −.08, p = .21).

**Table 4:** **Regression analysis: Intention and habit as predictors of unhealthy snack intake**

	**Step 1 β**	**Step 2 β**	**Step 3 β**
*Main analysis (n = 228)* †			
Intention	-.22**	-.17**	-.17*
Habit		.23**	.23**
Intention x habit			-.08
Model F	3.38**	4.78***	4.34***
R^2^	.07	.11	.12
R^2^ change		.04**	.006
*Sensitivity analysis 1 (n = 236)* †			
Intention	-.22**	-.17**	-.17*
Habit		.26***	.26***
Intention x habit			-.04
Model F	11.97**	15.06***	10.15***
R^2^	.05	.11	.12
R^2^ change		.07***	.002
*Sensitivity analysis 2 (n = 236)* †			
Attitude	.11	.15	.15
Subjective norms	.26***	.21**	.21**
Perceived behavioural control	-.12	-.07	-.07
Intention	-.35***	-.33**	-.33**
Habit		.22**	.22**
Intention x habit			-.05
Model F	7.93***	8.78***	7.43***
R^2^	.13	.16	.17
R^2^ change		.04**	.003

*p < .05, **p < .01, ***p < .001. Main analysis models adjust for demographics (coefficients not shown). Sensitivity analysis 1 models exclude demographics. Sensitivity analysis 2 models adjust for TPB variables, and exclude demographics. † Sample sizes differ across analyses due to missing data on demographic variables.

#### Sensitivity analyses

The same pattern of results held in models from which demographics were excluded, and those in which both demographics and TPB variables were controlled. When excluding demographics, within the regression model at the second step (R^2^ = .11, Model F[2,237] = 15.06, p < .001), intention (β = −.17, p = .01) and habit (β = .26, p < .001) predicted snack intake, but in the model at the third step (R^2^ = .12, Model F[3,236] = 15.06, p < .001), the intention x habit interaction did not (β = −.04, p = .52). Similarly, when controlling for TPB variables, within the regression model at the second step (R^2^ = .16, Model F[5,231] = 8.78, p < .001), intention (β = −.33, p = .001) and habit (β = .22, p = .001) predicted snack intake, but in the model at the third step (R^2^ = .16, Model F[6,230] = 7.43, p < .001), the intention x habit interaction did not (β = −.05, p = .39). Thus, no analysis supported Hypothesis 2.

## Discussion

Evidence of habit overriding intentions in guiding behaviour has come mostly from studies of concordant intentions and habits. This study explored whether counterintentional habits (for unhealthy snacking) would dominate over intentions (to eat unhealthy snacks) in directing behaviour (unhealthy snack intake). While habits were positively correlated with behaviour, contrary to habit theory, habits did not interact with intentions in predicting unhealthy snack intake. This questions a fundamental assumption around habitual health behaviour, and calls for further theorising around the precise role of habit in predicting health behaviour.

Our results question whether habits have the capacity to override intentions in producing behaviour. Previous evidence of such a relationship may be unreliable, because habits and intentions have been measured in the same direction (e.g. habitual snacking and intentions to snack; Danner et al. 2008), and have been highly correlated, making estimates of behaviour where habit is strong and intention weak lack validity (Gardner 2015a). It is possible that counterintentional habits may dominate over intentions in some settings, but our findings provide a negative case that is sufficient to show that this is not always the case. Indeed, our results mirror the few studies that have examined conflicting habits and intentions and found no interaction (Gardner et al. 2012b; Murtagh *et al.*^2012^; Verplanken and Faes 1999). There is no reason to expect that our findings lack validity, as we used similar methods to those used in studies in which an interaction has been found, with the only exception being that we measured conflicting, rather than congruent, habits and intentions, which better represents settings in which habit and intentions would be expected to prompt opposing behavioural patterns. Echoing findings from a meta-analysis of 21 studies in the dietary and physical activity domains, which found habit to correlate moderately-to-strongly with behaviour (Gardner *et al.*^2011^), habit was moderately positively correlated with snack intake, indicating that participants were more likely to snack where they had snacking habits. The expectation that habit will consistently override intention in directing behaviour is based on the assumption that habitual actions are largely uncontrollable in associated settings (Orbell and Verplanken 2010). Our data argue against this assumption, by indicating that intentions remained significantly and equally predictive of behaviour at all levels of habit; that is, people can act contrary to their habitual tendencies (e.g. Neal et al. 2013; Quinn et al. 2010). In one diary study, students reported the frequency with which they performed unwanted actions, and the methods that they used to inhibit them. Results indicated that vigilantly monitoring behaviour in settings that are conducive to habitual action and, to a lesser extent, distracting oneself, were effective for overriding the habit impulse (Quinn *et al.*^2010^). Habitual tendencies can therefore be inhibited (Gardner 2015b). This may be facilitated by self-control: a wealth of research suggests that people with greater self-control are less likely to engage in unhealthy behaviours, such as eating a high-fat diet (de Ridder et al. 2012; Wills et al. 2007). Temporal Self-Regulation Theory proposes that ‘prepotent’ default responses, such as those generated by habit, take precedence over alternative responses (e.g. intended responses) unless they are wilfully and effortfully resisted (Hall and Fong 2007). This predicts a three-way interaction between self-control, habit strength and intention, such that habit strength will overrule intentions only where self-control is weak, but where self-control is strong, the intention-behaviour relationship will be reinstated because prepotent habitual actions are consciously restrained. We did not measure dietary self-control in this study and so could not test this hypothesis. However, one study showed that, under conditions of high self-control, unwanted habits could be inhibited, but where self-regulatory capacity was diminished, people were less able to block their unwanted habits (Neal *et al.*^2013^). Neal et al’s findings undermine the suggestion that habits will always moderate the intention-behaviour relationship by showing that, where intention is accompanied by self-control, habitual action can be prevented. Indeed, some studies have shown that merely forming a counterhabitual intention may be sufficient to mobilise the self-regulatory resources needed to shield goal pursuit from the intrusion of an unwanted habit (Danner et al. 2011).

Our results have important implications for behaviour change interventions. It has previously been claimed, on the basis of the assumed dominance of habit over intention in guiding behaviour, that consciously motivating individuals to want to change their behaviour will be largely ineffective in shifting ingrained, habitual behaviour patterns (e.g. Verplanken and Wood 2006). If habit does not moderate the intention-behaviour relationship, then this claim may not be valid. Some evidence has shown that motivational interventions can have *greater* impact among those with strong habits: Eriksson et al. (2008) found that having car drivers complete a diary planning each of their coming journeys, and consider alternative transport options for each journey, was most effective in reducing car use among those with strongest self-reported driving habits. While breaking habits may be cognitively effortful and demanding (e.g. Neal *et al.*^2013^), it is nonetheless possible that intervention recipients with strong habits may make positive behaviour changes following a motivational intervention (Gardner 2015a).

Although no habit-intention interaction was found, both habits and intentions were significantly predictive of snack intake, with habitual snackers reporting higher snack intake, and those who intended to avoid snacking reporting lower snack intake. The significant, albeit small, negative correlation between intention and habit suggests that at least some participants had both snacking habits and intentions to avoid snacking. This likely reflects that, for these people, snacking was on some occasions habitually controlled, and on other occasions may have been mindfully inhibited. Sophisticated accounts of human motivation portray behaviour as the output of a chaotic struggle between in-situ facilitatory and inhibitory forces, such that people do whatever they most want or need to do *at any given moment* (West and Brown 2013). People with snacking habits and intentions to avoid snacking may be better able to inhibit their habitual tendencies on occasions where their intentions are particularly salient and self-regulatory capacity is strong, and less able where self-regulatory capacity (i.e., availability of attention and memory resources) is diminished or other goals are prioritised. The behaviour measure we used assessed how many times 21 possible snacks were consumed per day over a two-week period, and so represents an aggregate of a potentially huge number of discrete incidences of behaviour. We cannot therefore investigate the extent to which each of these instances was habitual or reasoned (e.g. Sniehotta 2009). The intention measure also assessed a global intention towards behaviour over the coming two weeks, but intentions can fluctuate over time and may not be remembered at the time of action (Einstein et al. 2003). These reflect crucial limitations of the data collection and analysis methods that dominate the habit field (Gardner 2015a); the effect of habits and intentions on the action of an individual on discrete occasions cannot be reliably estimated based on data aggregated across individuals and instances (e.g. Jaccard 2012). It is possible that habits do indeed dominate over intentions in regulating behaviour, but that the methods used herein, and which dominate the habit research field within social and health psychology, are insufficient to capture such effects. Methods that are more sensitive to discrete behaviour instances are available. Single-case designs, in which an individual reports his or her cognitions and behaviour over a period of time, are more suitable to scrutinising the ebb and flow of in-situ behaviour, and the potentially varying influence habits and intentions over time (Johnston and Johnson 2013). Technological advancements, such as the proliferation of smartphones, provide realistic opportunities to obtain rich real-time measures of in-situ cognitions and actions (Jones and Johnston 2011).

Limitations must be acknowledged. We modelled relationships between intentions and counterintentional habits in relation to diet, a domain in which we expected many conflicting intentions and habits. Yet, the small negative intention-habit correlation indicated that many participants did not hold directly opposed habits and intentions. It is possible that a true interaction may have emerged had habits and intentions more strongly conflicted. Future work might explore the role of counterintentional habits in the intention-behaviour relation more reliably by purposefully recruiting samples most likely to hold incongruent habits and intentions (e.g. new dieters), or examining behaviours likely to invite such conflict (e.g. habitual speeding versus intending to adhere to the speed limit). However, the lack of strong habit-intention conflict need not invalidate our findings. Where habit and intention correlate strongly and positively, predictions of action where habit is strong and intention weak can lack validity. A negative correlation, or no meaningful correlation, is most likely to indicate that participants either have opposing habits and intentions, or that intention strength varies independently of habit, in which case no such threat to validity is posed. Furthermore, measuring incongruent habits and intentions reduces the likelihood that participants will give similar answers to both sets of questions due to not recognising the distinction between them, which may render results more reliable (Ogden 2003).

It is also possible that a true habit-intention interaction was not detected by our self-report habit measure. Concerns have been raised around the accuracy of reflections on automatic processes (Hagger et al. 2015; but see Orbell and Verplanken 2015), and self-reports of unhealthy habits can also be biased by self-presentation concerns (Gardner and Tang 2014). It has been suggested that habit may be more reliably revealed by measures of performance frequency in stable contexts (Labrecque and Wood 2015). Yet, these assess the likelihood that habit has formed, rather than habit strength, and may conflate habitual and non-habitual frequent action (Gardner 2015a). The automaticity-specific index used in this study is theoretically and practically optimal for survey-based research (Gardner 2015a).

Behaviour was also measured via self-report, which can underestimate engagement in unhealthy behaviour (e.g. Hebert *et al.*^1997^). The extent to which participants could accurately recall their intake of discrete snacks over the preceding two-week period may also be questioned (Livingstone *et al.*^2004^). However, among adults, comparisons with objective dietary intake have shown self-reported diet measures to have a high degree of accuracy (Conway et al. 2004), and the extensive piloting of our snacking intake measure is likely to have increased validity. Additionally, participant recall of food consumption and self-estimated portion sizes have been used reliably in previous research (Kennedy et al. 1995; Guenther et al. 2008). Nonetheless, the aim of our study was to use methods similar to those in which habit has been shown to moderate the intention-behaviour relationship. In this respect, limitations inherent to our study are likely to have equally affected previous studies that found the expected moderation effect, which have been based on self-report behaviour and cognition measures (see Gardner *et al*. 2011).

## Conclusions

Our study suggests that habits do not necessarily override the influence of intention on behaviour, and that previous evidence purportedly showing this effect may be methodologically flawed due to the measurement of congruent and strongly correlated habits and intentions. If habits do not dominate over intentions in regulating behaviour, then previous claims that changing motivation will be insufficient for changing habitual behaviour may be premature. More sophisticated data collection and analysis methods may aid efforts to capture the momentary influence of habits and intention within individuals.

## Additional files

Additional file 1:
**Time 1 questionnaire.**
Additional file 2:
**Time 2 questionnaire.**

## Abbreviations

BMI: Body mass index
PBC: Perceived behavioural control
SD: Standard deviation
T1: Time 1
T2: Time 2
TPB: Theory of planned behaviour
UK: United Kingdom
