# Supplementary material for: Do habits always override intentions? Pitting unhealthy snacking habits against snack-avoidance intentions
Source: BMC Psychol. 2015 Mar 24;3(1):8. doi: 10.1186/s40359-015-0065-4 (PMC4374191; doi:10.1186/s40359-015-0065-4)
Supplement: Additional file 1: — Time 1 questionnaire. [file 40359_2015_65_MOESM1_ESM.docx]

Completion should only take **10­15 minutes**.

Taking part is completely voluntary and you are free to withdraw at any time without giving reason, and without penalty. You are free to not answer any questions you do not want to and will be able to exit the survey at any time. Your responses will be confidential and once both questionnaires are submitted to the researcher, personal information will be removed. This means that it will not be possible to withdraw data from the study from this point onwards.

Followed by 7 options from 'disagree strongly' to 'agree strongly' for you to select the one that is most appropriate to you.

*"I intend to avoid eating unhealthy snacks over the next two weeks"*

**What will I be asked to do?**

First we ask a few questions about you (such as occupation) and then ask you to tick a box in response to a series of statements such as the following:

**Why is this study being done?**

We would like to understand more about people’s snacking behaviour so we can provide valuable information for healthcare professionals to design programmes to help people change their eating behaviours and improve their health.

**Prize draw**

As a thank you for fully completing both parts of the study, you will be given the option in part 2 to enter a prize draw to win **£50** in vouchers to spend at [Amazon.co.uk](http://Amazon.co.uk).

We would like to invite you to take part in our 2­part research project which looks at adults’ thoughts and feelings about eating snacks.

You will be asked for your email address so we can send you a second, much shorter, questionnaire in two weeks. This second questionnaire is very important and will only take 6­-8 minutes to complete. Your email address will not be stored with your responses so your data will not be identifiable.

**Exploring adults’ thoughts and feelings towards snacking**

-

**Part 1**

***This questionnaire asks you about your thoughts and feelings about snacking.
Whatever your snacking habits might be, we would like to hear from you!***

**THANK YOU VERY MUCH FOR YOUR HELP**

This study has been approved by the UCL Research Ethics Committee [*reference number quoted*].

**What if I have any questions?**

If you would like more information about weight issues or eating behaviour, you can visit [www.weightconcern.org.uk](http://www.weightconcern.org.uk) or contact your GP. If you have any questions about this study or would like to receive a summary of our findings, please contact the researcher, [*researcher name and email address provided*]

The only personal information you will be asked to provide is an email address so that we can send you the second part of the study in two weeks’ time, and so we may enter you to the prize draw if you would like us to. It will not be possible to identify your specific questionnaire data at a later stage as any identifying details will be removed and stored separately once both parts have been submitted to us.

completing and returning the questionnaire, you are giving consent for the personal

information you provide to be used only for the purpose of this project and not transferred to an organisation outside of UCL. Your information will not be passed to any third parties. The information will be treated as strictly confidential and handled in accordance with the provisions of the Data Protection Act 1998.

**What will happen to my information?**

Your answers, and any other information you provide, will be completely confidential. By

**Exploring adults’ thoughts and feelings towards snacking**

-

**Part 1**

**Exploring adults’ thoughts and feelings towards snacking**

-

**Part 1**

Please answer the following questions about yourself

Male

Female

Current age

Date of birth

(DD/MM/YYYY)

Feet

Inches

Centimetres

Stones

Pounds

(lbs)

Kilograms

(kg)

White­British

Black­African

Bangladeshi

White­Irish

Black­Other

Asian Other

White­Other

Indian

Chinese

Black­Caribbean

Pakistani

Other (please specify)

***1. Are you male or female?**

**3. What is your current age and date of birth?**

***2. Please enter your email address in the box below**

**It would be helpful for our research team to know your height and weight**

**4. How tall are you?**

**5. OR**

**6. How much do you weigh?**

**7. OR**

**8. To which ethnic group do you consider you belong?**

**Exploring adults’ thoughts and feelings towards snacking**

-

**Part 1**

No educational qualifications

'A' or 'AS' Level/Higher School

CSE, GCSE or 'O' Level

Vocational qualifications (e.g. NVQ1&2)

Other (please specify)

A home

A private

A council

Living with

owner

tenant

tenant

parent/relative

Yes

No

If yes please write your occupation here

**9. What is your highest level of education?**

**10. Are you:**

**11. Are you currently employed?**

Undergraduate degree

Postgraduate qualification (e.g. Masters, PhD)

Certificate (e.g. NVQ3)

**Exploring adults’ thoughts and feelings towards snacking**

-

**Part 1**

A **snack** is considered something **eaten** which is **not part of a meal**.

**next two weeks is**

**13. My attitude towards avoiding eating unhealthy snacks over the**

**snacks over the next two weeks**

**14. I have complete control over whether I avoid eating unhealthy**

**12. I intend to avoid eating unhealthy snacks over the next two weeks**

**Exploring adults’ thoughts and feelings towards snacking**

-

**Part 1**

Please select the response that is most appropriate to you.

We use the term **"unhealthy"** throughout the survey to mean snacks that are high in fat, sugar or salt according to UK guidelines.

Disagree

Disagree

Disagree

Neither

Agree

Agree

Agree

strongly

slightly

agree nor

slightly

strongly

disagree

Extremely

Quite

Slightly

Neither

Slightly

Quite

Extremely

negative

negative

negative

positive or

positive

positive

positive

negative

Disagree

Disagree

Disagree

Neither

Agree

Agree

Agree

strongly

slightly

agree nor

slightly

strongly

disagree

**15. People who are important to me think I should avoid eating unhealthy snacks over the next two weeks**

Disagree strongly

Disagree

Disagree

Neither

Agree

Agree

Agree

slightly

agree nor

slightly

strongly

disagree

**19. The people in my life whose opinion I value would approve of me**

**next two weeks is**

**20. I expect to avoid eating unhealthy snacks over the next two weeks**

**avoiding eating unhealthy snacks over the next two weeks**

**avoid eating unhealthy snacks over the next two weeks?**

**16. I want to avoid eating unhealthy snacks over the next two weeks**

**Exploring adults’ thoughts and feelings towards snacking**

-

**Part 1**

Disagree

Disagree

Disagree

Neither

Agree

Agree

Agree

strongly

slightly

agree nor

slightly

strongly

disagree

**17. My attitude towards avoiding eating unhealthy snacks over the**

Extremely

Quite

Slightly

Neither

Slightly

Quite

Extremely

unfavourable

unfavourable

unfavourable

favourable

favourable

Favourable

favourable

nor

unfavourable

**18. How much control do you think you have over whether or not you**

No

Slight

A

Moderate

Quite

A

Complete

control

control

little

control

a lot of

lot of

control

control

control

control

Disagree

Disagree

Disagree

Neither

Agree

Agree

Agree

strongly

slightly

agree nor

slightly

strongly

disagree

Disagree

Disagree

Disagree

Neither

Agree

Agree

Agree

strongly

slightly

agree nor

slightly

strongly

disagree

**23. Eating unhealthy snacks is something I do without thinking**

**consciously remember**

**22. Eating unhealthy snacks is something I do without having to**

**21. Eating unhealthy snacks is something I do automatically**

5 6

5 6

5 6

5 6

**Exploring adults’ thoughts and feelings towards snacking**

-

**Part 1**

Using the 1 to 7 scale below, please indicate how much each of the following statements reflects how you typically are

1

2

3

4

7

(strongly

(strongly

disagree)

agree)

1

2

3

4

7

(strongly

(strongly

disagree)

agree)

1

2

3

4

7

(strongly

(strongly

disagree)

agree)

**24. Eating unhealthy snacks is something I start doing before I realise I'm doing it**

1

2

3

4

7

(strongly

(strongly

disagree)

agree)

**Exploring adults’ thoughts and feelings towards snacking**

-

**Part 1**

We will send you a second, shorter questionnaire in approximately two weeks' time which simply asks you a few further items. **This second questionnaire is very important and will only take 6­8 minutes to complete.**

**What if I have any questions?**

If you would like more information about weight issues or eating behaviour, you can visit

[www.weightconcern.org.uk](http://www.weightconcern.org.uk) or contact your GP. If you have any questions about this study or would like to receive a summary of our findings, please contact the researcher, [*name and email address given*]

**25. Please re­enter your email address below in order that the second short questionnaire can be sent to you in 2 weeks**

**Thank you for taking the time to complete the first questionnaire**
