# Supplementary material for: Do habits always override intentions? Pitting unhealthy snacking habits against snack-avoidance intentions
Source: BMC Psychol. 2015 Mar 24;3(1):8. doi: 10.1186/s40359-015-0065-4 (PMC4374191; doi:10.1186/s40359-015-0065-4)
Supplement: Additional file 2: — Time 2 questionnaire. [file 40359_2015_65_MOESM2_ESM.doc]

//

**Exploring adults' thoughts and feelings towards&nbsp;snacking** - **Part** 2

Welcome to the final part of a 2­part survey. Please complete this very brief survey (6­8 minutes) if you completed a survey titled **‘Exploring adults' thoughts and feelings towards snacking ­ Part 1’** approximately 2 weeks ago.

This survey is very important to the research project so please complete the information as accurately as possible. All responses will be held anonymously when stored.

The following questions ask you about the **type** and **amount** of snacks you have consumed **per week**, over the past **2 weeks.**

You will be given the option to enter our **prize draw** to win £50 in vouchers to spend at [Amazon.co.uk](http://Amazon.co.uk/) at the end of the survey.

***1. Please enter below the email address you gave in the first questionnaire**

**2. Please give your date of birth to allow us to match your responses with the part 1 questionnaire**

DD

MM

YYYY

Date of

Birth

**Exploring adults' thoughts and feelings towards&nbsp;snacking** - **Part** 2

The following questions ask you about the **type** and **amount** of snacks you have consumed either daily, per week, or not at all over the past **2 weeks.**

Please read the following instructions carefully.

l Please indicate how frequently you consumed the snacks in the list below in the **PREVIOUS TWO WEEKS.**

l A **snack** is considered something **eaten** which is **not part of a meal**. There is also space for you to tell us about any other snacks you consumed that are not listed.

l We ask you to think about the portion size of your typical snacks. An example of a

medium portion is described for each food (in brackets after the food name). Your portion may be bigger or smaller than a medium portion. Please decide if your typical portion is small (i.e. half the medium portion described), medium (as described), large (double the portion described) or extra­large (more than double the portion described).

l We also ask you to tell us whether you would consider each snack to be healthy or unhealthy, regardless of the amount consumed.

**Exploring adults' thoughts and feelings towards&nbsp;snacking** - **Part** 2

**3. Please complete ALL columns of the table – if you didn’t consume the food option shown there is a response to indicate 'none'**

Frequency of

Do you consider this

consuming each

Typical portion size
consumed

snack to be healthy or

snack over the past 2

unhealthy?

weeks

Fresh fruit (Medium

serving = 1 piece eg;

apple or small bowl eg;

berries)

Dried fruit (Medium


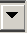


serving = Handful/2

tablespoons)

Chocolate (Medium

serving = Regular sized

bar approx 50g)

Crisps (Medium serving
1 small 25g bag)

Nuts & seeds unsalted

(Medium serving

Handful, 20 almonds or 10 brazil nuts)

Nuts salted, flavoured

or coated (Medium

serving = Handful/25g

bag)

Biscuits

plain (Medium

serving

2 small

biscuits)

Biscuits ­ chocolate or

cream (Medium serving

2 small biscuits)

Crackers and savoury

biscuits (Medium

serving = 2

crackers/biscuits)

Breadsticks, oatcakes,

pretzels (Medium

serving = 2

pieces/slices)

Rice cakes (Medium

serving = 2

pieces/slices)

Cheese (Medium serving
3 thin slices, approx

**Exploring adults' thoughts and feelings towards&nbsp;snacking** - **Part** 2

30g)

Toast or bread (Medium

serving = 2 medium

slices)

Butter/margarine/spread

(used on crackers/bread

etc) (Medium serving

2 teaspoons/10g)

Cakes & sweet pastries

(Medium serving = 1

cake, eg; muffin or

pastry, or 1 medium

slice)

Yogurt (Medium serving

single portion pot,

120g)

Raw vegetables

(Medium serving

5 or

6 sticks/batons eg;1

whole carrot)

Dips (eg; houmous or

salsa) (Medium serving

2 tablespoons/30g)

Sweets (Medium serving

1 small packet, eg;

tube of fruit pastilles)

Savoury pastries (eg:

pasties, sausage rolls)

(Medium serving = 1

piece, eg; sausage roll

approx 60g)

Cereal bars (Medium serving = 1 bar)

Other 1

Other 2

Other 3

Other 4

Other 5

Where you have completed 'Other' above, please list the type of snack listed from 1­5. There is space for additional comments if you need to tell us more.

**5**

**Exploring adults' thoughts and feelings towards&nbsp;snacking** - **Part** 2

**6**

**Exploring adults' thoughts and feelings towards&nbsp;snacking** - **Part** 2

All participants completing both part 1 and part 2 of the survey are eligible to enter our prize

draw.

Please indicate below if you would like to be entered to win a £50 voucher to spend at [Amazon.co.uk](http://Amazon.co.uk/).

***4. I wish to be entered to the prize**

**draw. I have provided a valid email address below so that I can be notified should I win.**

jlknm Yes

jlknm No

If yes, please enter your email address below:

Prize Draw

**Exploring adults' thoughts and feelings towards&nbsp;snacking** - **Part** 2

**Thank you for taking the time to participate in this research**

**What if I have any questions?**

If you would like more information about weight issues or eating behaviour, you can visit [www.weightconcern.org.uk](http://www.weightconcern.org.uk/) or contact your GP.

If you have any questions about this study or would like to receive a summary of our findings, please contact the researcher, Sharon Corbridge at [s.corbridge.12@ucl.ac.uk](mailto:s.corbridge.12@ucl.ac.uk)
